# First evaluation of antibody responses to *Culex quinquefasciatus* salivary antigens as a serological biomarker of human exposure to *Culex* bites: A pilot study in Côte d’Ivoire

**DOI:** 10.1371/journal.pntd.0010004

**Published:** 2021-12-13

**Authors:** Bi Zamble H. Zamble, Serge S. Yao, Akré M. Adja, Mahfoud Bakli, Dounin D. Zoh, Françoise Mathieu-Daudé, Serge B. Assi, Franck Remoue, Lionel Almeras, Anne Poinsignon

**Affiliations:** 1 Institut Pierre Richet / Institut National de Santé Publique, Bouaké, Côte d’Ivoire; 2 MIVEGEC, University of Montpellier, IRD, CNRS, Montpellier, France; 3 Institut Pasteur de Côte d’Ivoire, Abidjan, Côte d’Ivoire; 4 UFR Biosciences, University Felix Houphouët Boigny, Abidjan, Côte d’Ivoire; 5 IHU Méditerranée Infection, Marseille, France; 6 Programme National de Lutte contre le Paludisme, Abidjan, Côte d’Ivoire; 7 Unité Parasitologie et Entomologie, Département Microbiologie et Maladies Infectieuses, Institut de Recherche Biomédicale des Armées, Marseille, France; 8 Aix Marseille Univ, IRD, SSA, AP-HM, VITROME, Marseille, France; National Institutes of Health, UNITED STATES

## Abstract

**Background:**

*Culex* mosquitoes are vectors for a variety of pathogens of public health concern. New indicators of exposure to *Culex* bites are needed to evaluate the risk of transmission of associated pathogens and to assess the efficacy of vector control strategies. An alternative to entomological indices is the serological measure of antibodies specific to mosquito salivary antigens. This study investigated whether the human IgG response to both the salivary gland extract and the 30 kDa salivary protein of *Culex quinquefasciatus* may represent a proxy of human exposure to *Culex* bites.

**Methodology/Principal findings:**

A multidisciplinary survey was conducted with children aged 1 to 14 years living in neighborhoods with varying exposure to *Culex quinquefasciatus* in the city of Bouaké, Côte d’Ivoire. Children living in sites with high exposure to *Cx quinquefasciatus* had a significantly higher IgG response to both salivary antigens compared with children living in the control site where only very few *Culex* were recorded. Moreover, children from any *Culex*-high exposed sites had significantly higher IgG responses only to the salivary gland extract compared with children from the control village, whereas no difference was noted in the anti-30 kDa IgG response. No significant differences were noted in the specific IgG responses between age and gender. Sites and the use of a bed net were associated with the level of IgG response to the salivary gland extract and to the 30 kDa antigen, respectively.

**Conclusions/Significance:**

These findings suggest that the IgG response to *Culex* salivary gland extracts is suitable as proxy of exposure; however, the specificity to the *Culex* genus needs further investigation. The lower antigenicity of the 30 kDa recombinant protein represents a limitation to its use. The high specificity of this protein to the *Culex* genus makes it an attractive candidate and other specific antibody responses might be more relevant as a biomarker of exposure. These epidemiological observations may form a starting point for additional work on developing serological biomarkers of *Culex* exposure.

## Author summary

The evaluation of exposure to mosquitoes is a key parameter in assessing the risk of transmission of associated pathogens, including zoonoses. Entomological methods represent the gold standard but have several limitations, and efforts are being made to develop new indicators to accurately assess human–*Culex* contact. This study showed the IgG response to *Culex quinquefasciatus* salivary gland extract is suitable proxy of exposure to *Culex* bites. The lower antigenicity of the 30 kDa recombinant protein represents a limitation to its use. The high specificity of this protein to the Culex genus makes it an attractive candidate and other isotypic antibody responses specific to this salivary antigen might be more relevant as a biomarker of exposure.

## Introduction

*Culex spp*. mosquitoes (Diptera: Culicidae) are widespread globally, except in Antarctica. They can be found in tropical and temperate areas, with more than 770 species described and grouped into 26 subgenera. They can feed both on humans and animals [1,2] increasing their potential for transmission of zoonotic diseases and thereby making them a real threat to public health. *Culex quinquefasciatus* is the most common mosquito species in urban tropical settings [3] and is responsible for the transmission of a wide variety of pathogens, such as filaria parasites including *Wuchereria bancrofti* that cause lymphatic filariasis [4], and avian malaria parasites (*Plasmodium relictum*). It can also transmit numerous human viruses, including West Nile, Japanese encephalitis, Saint Louis, and Rift Valley fever viruses [5]. Moreover, even in the absence of disease transmission, these mosquitoes are a serious nuisance as biting pests worldwide [6].

*Culex quinquefasciatus* mosquitoes are widespread in Côte d’Ivoire [7] and a high seroprevalence of West Nile virus (WNV) has been reported in horses [8]. Human lymphatic filariasis is endemic in many districts [7] where mass drug administration has been introduced since 2014 to stop the spread of the infection, as recommended by the World Health Organization [9]. Thus, in the absence of human vaccines against most of the *Culex*-borne diseases and the difficulties to cover all endemic areas with drug treatment, disease transmission could be prevented through the reduction of *Culex* populations. The control of vector population consists of personal (e.g., long-lasting insecticide-treated mosquito nets) and collective strategies (e.g., improvement of the living environment, indoor residual spraying of insecticides) or innovative approaches such as the radiation-based sterile insect technique [10].

The most common approaches for evaluating the risk of disease transmission and the effectiveness of vector control strategies are based on entomological methods [11,12]. However, they have some limitations because they are labor-intensive, face budgetary and logistical constraints, and have ethical limitations when human-landing catches (HLCs) are deployed [13]. Although HLCs are the current relevant indicators of the intensity of contact between humans and vectors, these measures are generally applied to a limited area/population and do not take into account the heterogeneity of inter-individual exposure. Therefore, there is a demand for the development of alternative strategies to accurately evaluate human exposure to *Culex* mosquito bites at individual level, and to estimate the potential risk of *Culex*-borne pathogen transmission in exposed populations.

During blood-feeding, concomitantly to blood intake, female mosquitoes inject saliva containing a cocktail of biologically active proteins that counteract host homeostasis and modulate the vertebrate immune response [14,15]. The injection of vector saliva can also elicit a host antibody response against some salivary proteins and an interesting approach exploits the immunological properties of mosquito saliva in order to develop serological biomarkers of exposure to mosquito bites [16–19]. However, the use of whole saliva as an antigen source presents numerous limitations. Saliva collection or salivary gland dissection is tedious and time-consuming work, and the composition of salivary proteins (nature and amount) varies depending on the mosquito age and the time since blood-feeding [20,21]. Additionally, some salivary proteins are ubiquitous in various vector genera [22–25], which can lead to human antibody cross-reaction. Thus, it is necessary to identify antigenic salivary proteins specific to a genus or with low homology to other blood-sucking arthropods. Previous work combining immunoproteomic studies and bioinformatic predictions led to the identification of salivary proteins or peptides specific to main mosquito vectors. Specific immunoglobulin G (IgG) antibody responses to these salivary components have been validated as a serological biomarker of exposure to *Anopheles* (IgG response to gSG6 or CE5 salivary proteins, or to the gSG6-P1 salivary peptide), the vector of human malaria *Plasmodium* [26,27], and for *Aedes*, the vector of arboviruses (IgG to Nterm34-kDa, a salivary peptide) [28].

The immunogenicity of *Culex* spp. saliva has been a topic of interest, but most studies focused on identifying the main immunogenic salivary proteins responsible for allergy so as to develop high-sensitivity diagnostic tests and immunotherapy [29,30]. Studies identified *Cx*. *quinquefasciatus* salivary proteins that elicit an IgE and IgG responses in humans, with the major antigens being the D7-related proteins[30–33].

Only few studies have assessed human antibody responses to *Culex* salivary gland extract (SGE) in relation to exposure to *Cx*. *quinquefasciatus* or *Cx*. *pipiens*. Individuals living in region endemic for filariasis where *Cx*. *quinquefasciatus* was predominant had higher IgG and IgE titers than people living in a region where *Culex* was less prevalent, and the specific IgG level increased with age [34]. Another study had reported a seasonal dynamics in anti- SGE IgG response in an urban setting where *Culex* are predominantly anthropophilic, with the highest level during the warm season [35]. More studies are therefore needed to confirm whether specifc IgG responses to salivary gland extracts (SGE) may represent a proxy of exposure to *Culex* bites. It is also necessary to identify a specific salivary candidate to develop a serological tool for assessing specifically the exposure to *Culex* bites and thus the risk of transmission of associated pathogens. Few data on the *Culex* sialotranscriptome, including *Cx*. *pipiens*, *Cx*. *tarsalis*, and *Cx*. *quinquefasciatus*, have been published [22,36,37] that enable the identification of species-specific or genus-specific proteins.

In this study, we selected and tested the 30 kDa salivary gland allergen (gi|170033701) as a pertinent candidate marker of exposure to *Cx*. *quinquefasciatus* bites. This protein belongs to the 30 kDa antigen/GE-rich/Aegyptin family of proteins that is abundant in the salivary glands of adult anopheline and culicine female mosquitoes [37] including *Cx*. *quinquefasciatus* [22], *Aedes (Ae*.*) aegypti* [38], and *Anopheles (An*.*) gambiae* [39]. Members of this protein family have anti-homeostatic properties in binding collagen and preventing platelet aggregation [18,40]. They were reported to be highly antigenic in inducing an IgE response in humans exposed to *Aedes* species [30] and an IgG response in vertebrates exposed to *Culex* bites [32,33]. Previous bioinformatics work showed a low sequence identity between homologous members of the 30 kDa antigen family, suggesting weak cross-reactivity of human IgG between the related salivary proteins [40].

This study aimed to evaluate by immuno-epidemiological approach, whether the IgG antibody levels against *Cx*. *quinquefasciatus* SGE and the 30 kDa salivary gland recombinant allergen are suitable serological biomarkers of exposure to *Culex* bites. A multidisciplinary survey was conducted with children living in areas with various exposure to *Cx*. *quinquefasciatus* estimated by classical entomological indices in the city of Bouaké, Côte d’Ivoire. The sequence homology of the 30 kDa salivary gland allergen protein of *Cx*. *quinquefasciatus* was also assessed with its different orthologs in the Arthropoda phylum to define the specificity of this candidate.

## Methods

### Ethics statement

The present study followed the ethical principles recommended by the Edinburgh revision of the Declaration of Helsinki and was approved by the Ethics Committee of the Côte d’Ivoire Ministry of Health (June 2014; No. 41/MSLS/CNER-dkn). Site leaders provided prior permission to survey on each site and written informed consent of all parents or guardians of children who participated in the study was obtained before inclusion.

### Study sites

**Fig 1:**
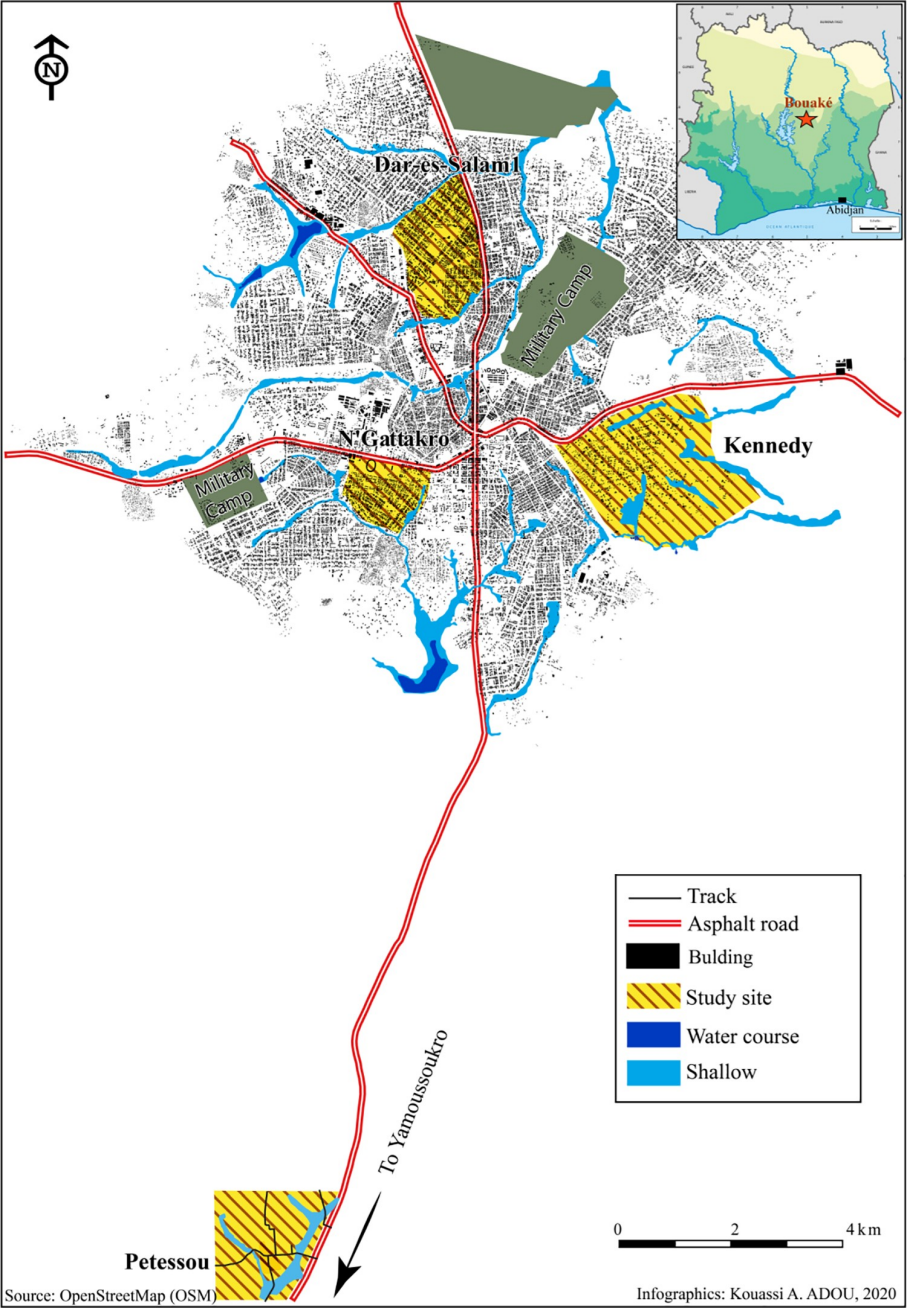
Map of study areas in the city of Bouaké, Côte d’Ivoire. Different study sites are hatched. Map source: https://www.openstreetmap.org/export#map=12/7.6821/-5.0039.

The study was carried out in the city of Bouaké (7°41N, 5°01W), located approximately 350 km North from Abidjan, Cote d’Ivoire (Fig 1). The study area and study design have been previously described in detail [41,42]. Briefly, the climate is tropical humid with two seasons: the dry season runs from November to March, and the rainy season occurs from April to October. The rainy season is marked by two maximum rainfalls, one in June and one in September, with an average annual rainfall of between 1,000 and 1,600 mm.

### Study population

The initial cohort consisted of 508 children aged from 6 months to 14 years from 5 sites and enrolled in a cross-sectional study which was carried out during the end of the dry season (March-April 2015). Households and children were randomly selected and socio-demographic (gender, age, sleeping or not under Insecticide-Treated Nets [ITNs]), geographical, entomological, and clinical data were collected. The present study was carried out on a sub-sample of the initial cohort and consisted of 223 children aged from 12 months to 14 years from 4 sites, Dar-es-Salam, N’Gattakro, Kennedy (three neighborhoods of Bouaké city), and Petessou (a village near Bouaké).

For immunological assays, blood samples were collected at the fingertips in microtainer tubes (microvette 500 serum-Gel Starstedt, Marnay, France) and sera were obtained after centrifugation at 3,000 rpm for 10 min. Sera were fractionated into aliquots and then frozen at -20°C until used.

### Mosquito collection

Adult mosquitoes were collected in March 2015, as previously described [41,43]. In each of the four sites, six catching points, three indoor and three outdoor were used to collect mosquitoes by landing catches on adult volunteers for two consecutive nights (from 6.00 pm to 06.00 am). Twelve adult mosquito catchers gave prior informed consent and received yellow fever vaccination and anti-malarial chemoprophylaxis as recommended by the National Malaria Control Program of Côte d’Ivoire. Adult mosquitoes were collected, counted and their species were morphologically classified at the laboratory. The human biting rate (HBR) of each mosquito species was calculated as the average number of mosquitoes collected per person per night (bites/human/night, BHN).

### Collection of *Culex quinquefasciatus* salivary gland extracts

Salivary gland extracts (SGE) were obtained from 8-day-old uninfected female *Cx*. *quinquefasciatus* (n = 673) reared in an insectary (Institut Pierre Richet, Bouaké, Côte d’Ivoire). Eggs were obtained from the Vectopole facility from Montpellier. Two days after a blood meal, the mosquitoes were sedated with cold and then their salivary glands were dissected out and transferred into a tube containing 30 μl of phosphate-buffered saline (PBS, Gibco tablet) and 5 μl of cocktail protease inhibitor (Sigma, St.-Louis, MO, USA). The dissected salivary glands were then pooled in 50 pairs per batch and frozen at −80°C before protein extraction. To disrupt the salivary gland membranes, three successive freeze-thaw cycles were done and the soluble salivary gland extract (SGE) fraction was then separated by centrifugation for 20 min at 30 000 g at +4°C. The protein concentration was evaluated in the supernatant by the Bradford method (OZ Biosciences, Marseille, France) after pooling the different gland batches to generate a homogenous SGE for immunological assessment. The final SGE pool containing 208 μg/mL of proteins was stored at −80°C before use.

### Expression and purification of the 30 kDa recombinant protein

The coding sequence of 30 kDa salivary gland allergen Aed a3 isoform X1 (gi|170033701) from *Culex quinquefasciatus* was retrieved from National Center for Biotechnology Information (NCBI) database. The cDNA of this selected protein was synthesized by Genecust (Genecust, Dudelange, Luxembourg) with a C-terminal His-tag and cloned into *E*. *coli* expression vector pET28b (Novagen). The fidelity of the cloned sequences was verified by DNA sequencing, using an ABI Prism 3100 analyzer (Applied Biosystems). The expression plasmids pET28b containing sequenced genes were transformed into E-coli BL21 (DE3) competent cells (Invitrogen). Bacterial precultures were performed overnight and grown in LB Broth Miller medium (Fisher Scientific) at 37°C with shaking (220 rpm). Fifty (50) mL of the saturated culture were then transferred into 5 L of LB Broth Miller medium and grown up to 0.8 OD_600_ before starting induction by IPTG (0.1 mM). After 4 hours of incubation at 30°C and shaking (220 rpm), cells were harvested (1,000×g for 30min at 4°C) and resuspended in 200 mL of lysis buffer containing 50 mMTris-HCl pH 8.0, 300 mM NaCl, and 10 mM Imidazole and sonicated on ice for 5 min. Cell debris was pelleted by centrifugation at 13,000×g for 1 hour at 4°C. The supernatants were filtered through 0.45 μM Durapore filters (Millipore, Bedford, MA, USA).

The recombinant 30 kDa protein presents in the supernatant, was purified under native conditions according to the manufacturer’s instruction using HisTrap HP columns (AKTA purifier 10 GEH, GE Healthcare, France). The 30 kDa protein was loaded with an Aekta fast protein liquid chromatography (LC; GE Healthcare, Glattbrugg, Switzerland) onto a His-Trap HP 5-mL column (GE Healthcare, Cat. No. 17-5248-02), which was equilibrated with Buffer A (50 mM Tris-HCl pH 8.0, 300 mM NaCl, and 10 mM Imidazole). After protein binding, the column was washed with 10 column volumes of Buffer A and then re-equilibrated with Buffer B (50 mM Tris-HCl pH 8.0, 300 mM NaCl, and 500 mM Imidazole). Bound proteins were eluted with Buffer B. The fractions containing the His-tagged recombinant proteins were selected based on the profile obtained by SDS-PAGE and were then pooled. To eliminate contaminant proteins, pooled fractions of each recombinant protein were further purified by gel filtration on HiLoad 26/60 Superdex 75 pg (GE Healthcare) gel filtration column in buffer containing 50 mMTris-HCl pH 8.0, 300 mM NaCl.

After protein purification, fractions containing the 30 kDa protein were collected and concentrated using a Centricon of 5 kDa cut*-*off (Amicon, USA) and then stored at 4°C. The 30 kDa protein concentration was measured using a Lowry DC Protein assay (Bio-Rad, Hercules, CA, USA).

### SDS-PAGE, In-gel digestion, and mass spectrometry analysis

The purities of purified extract were assessed by SDS-PAGE, and 30 kDa recombinant protein position was verified by immunoblot using an anti-His-Tag antibody (1/5000) (Invitrogen), as previously described [44].The identity of the respective detected band was confirmed by mass spectrometry (MS) as previously described [45]. Briefly, 5 μg of the purified recombinant protein was reduced in a Tris buffer containing dithiothreitol (1% w/v, Sigma), boiled for 5 min, and loaded onto a 12% polyacrylamide gel before being separated using a Mini PROTEAN II (Bio-Rad, Hercules, CA, USA). After electrophoresis, gels were stained with Coomassie brilliant blue R-250 (Imperial Protein Stain, Thermo scientific) and scanned with a high-resolution densitometer scanner (Image Scanner 3, GE Healthcare) and densitometry profiles were analyzed using the ImageQuant TL software (GE Healthcare). Protein bands from gels were excised for further identification by mass spectrometry. Molecular weights were estimated by comparison with standard molecular weight markers (Bio-Rad).

Excised bands were digested overnight at 37°C with sequencing-grade trypsin (12.5 μg/mL; Promega Madison, WI, USA) in 50 mM NH_4_HCO_3_ (Sigma). The resulting peptides were extracted with 25 mM NH_4_HCO_3_ for 15 min, dehydrated with acetonitrile (Sigma), incubated with 5% acid formic (Sigma) for 15 min under agitation, then dehydrated with acetonitrile, and finally completely dried using a SpeedVac. The samples were then analyzed on a MALDI-TOF MS (Bruker Daltonics) for identification. The quantity of recombinant protein produced was estimated using a NanoDrop ND-1000 spectrophotometer (NanoDrop Technologies, Inc., Wilmington, DE). We also verified that the 30kDa salivary protein is the main immunogenic component of the recombinant solution. Western blots were carried out on sera from individuals naturally exposed to *Culex* bites who were previously tested by ELISA [35].

### Evaluation of human IgG level for *Cx*. *quinquefasciatus* SGE and the 30 kDa recombinant protein

IgG level to SGE and 30 kDa recombinant protein was measured by indirect ELISA. Ninety-six (96) well Maxisorp plates (Nunc, Roskilde, Denmark) were coated with SGE or the 30 kDa recombinant protein at a final concentration of 1 μg/ml in coating buffer (PBS) and incubated for 150 min at 37°C. Plates were blocked with 200 μL of protein-free Blocking Buffer (Thermo Scientific, Rockford, USA) and incubated for 60 min at 37° C. Individual sera were diluted in buffer (PBS-Tween 1%) and incubated at 4°C overnight at a final dilution of 1/200. Monoclonal mouse biotinylated anti-human IgG (BD Pharmingen, San Diego, CA) was added in at a 1/2000 dilution (PBS-Tween 1%) for 90 min at 37°C. Peroxidase-conjugated streptavidin (GE Healthcare, Orsay, France) was then added (1/1000 in PBS-Tween 1%) for 60 min at 37°C. Colorimetric development was carried out with 2,2’-azino-bis ethylbenzothiazoline 6-sulfonic acid) diammonium salt (sigma, Saint-Louis, MO, USA) in 50 mM citrate buffer (pH = 4 containing 0.003% H_2_O_2_) and absorbance (optical density [OD]) was measured at 405 nm (Multisakan GO Thermo Scientific). Each sample was tested in duplicate wells containing SGE or the 30 kDa protein and in a well without antigen to measure non-specific reactions. Individual results were expressed as the ΔOD value: ΔOD = ODx − ODn, where ODx represents the mean of the individual OD value in both wells with salivary antigen and ODn the individual OD value in a blank well containing no antigen.

### Protein sequence analysis

Arthropod protein sequences related to the 30 kDa protein sequence (gi|170033701; XP_001844715.1) of *Cx*. *quinquefasciatus* were retrieved from the NCBI using the BLASTp program. For each species, when several hits were found, sequences presenting more than 90% identity were excluded and the protein homolog producing the best score was chosen for further protein sequence comparisons between orthologs, *i*.*e*. percent identity and sequence alignment. The sequences of *Cx*. *tarsalis* orthologs identified previously [25] were retrieved manually from the NCBI nucleotide database. PSI-Blast iterations were run to retrieve all sequences with E-value below the threshold of 0.001. Multiple sequence alignment was performed using the Clustal Omega program (EMBL-EBI search and sequence analysis tools: www.ebi.ac.uk/Tools/msa/clustalo/) and the multiple alignment viewer MView 1.63 (EMBL-EBI).

### Statistical data analysis

Statistical analyzes were done using Graph Pad Prism 5 software (San Diego, CA) and R (Version 3.5.3, R Core Team, Vienna, Austria). After checking that our data did not follow a Gaussian distribution (normal distribution), the non-parametric Mann-Whitney test was used to compare the Ab levels between two independent groups and the non-parametric Kruskal-Wallis test used to compare the levels of Abs between more than two groups and the Dunn’s post-hoc test was also performed for two-by-two comparisons in more than three groups. The Spearman’s rank correlation test was used to analyze the correlation between the OD of tested sera with recombinant 30 kDa and total SGE in each high exposed study site to *Cx quinquefasciatus*. The human biting rate (HBR) was compared between sites by using the prop. test testing the null hypothesis that the proportions in several groups are the same.

Univariate analysis was conducted with each covariate, and multivariate linear regression analyses were performed with all covariates with a *p-value* set at < 0.20 in univariate analysis. Final models were adjusted by backward selection and removing non-significant variables at *p-value* > 0.05. Maximum likelihood methods were used to identify the best-fitting models according to AIC value (Akaike Information Criterion). All differences were considered significant at a *p-value* <0.05.

## Results

### Specificity of the 30 kDa salivary protein sequence in phylum Arthropoda

**Fig 2:**
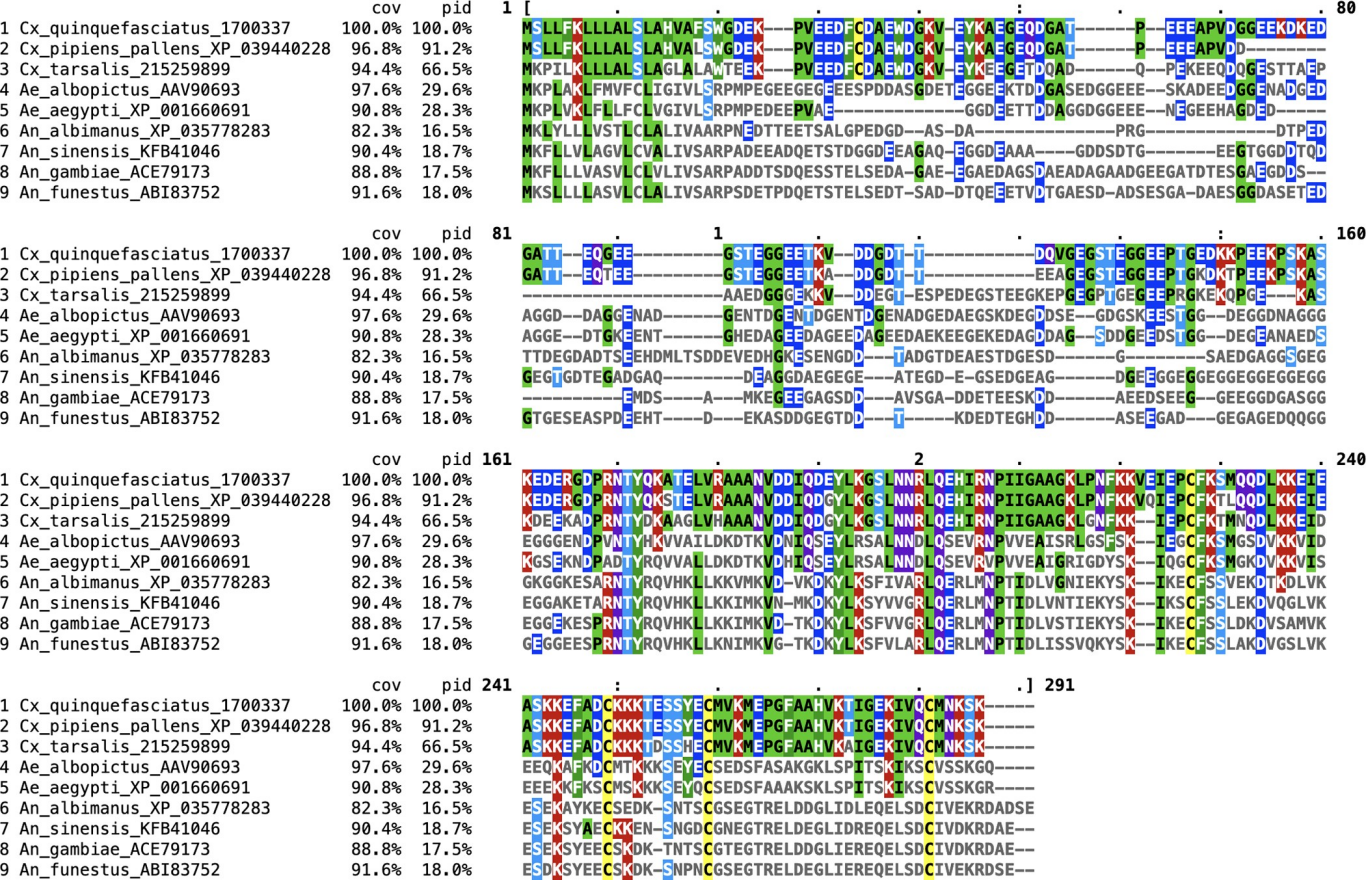
Alignment of arthropod protein orthologs of the 30 kDa protein from *Cx*. *quinquefasciatus*. Species, accession numbers, percentage of protein coverage (cov), percentage of identical amino acid residues (pid) and amino acid positions are indicated. Residues identical to the reference sequence of *Culex quinquefasciatus* are highlighted in different colors according to the amino acid properties. For each species the protein homolog producing the best score was selected for the alignment.

A BLASTp search for the 30 kDa protein (gi|170033701) in the NCBI database retrieved the orthologous sequences in different species of the phylum Arthropoda. All sequences displaying a significant E-value (<0.001) were proteins of mosquito species belonging to the *Culicidae* family. Among them, *Cx*. *pipiens pallens* and *Cx*. *tarsalis* orthologs showed high sequence identity (91% and 68%, respectively) and high protein coverage (96% and 92%, respectively) with the *Cx*. *quinquefasciatus* protein sequences. All the other proteins encoded in the phylum Arthropoda displayed a lower sequence identity (≤ 39%). Outside the *Culex* genus, proteins presenting the highest identities were from *Ae*. *albopictus* and *Ae*. *aegypti* (with 36% and 39% identity, and 76% and 49% for coverage, respectively), whereas the orthologs found in *Anopheles* species displayed a lower identity (<34%). Alignment of the orthologous sequences of the *Cx*. *quinquefasciatus* 30 kDa protein in each species presenting the best score value is presented in Fig 2. Using Clustal Omega, the whole sequence of the proteins was considered for the calculation of the alignment cover and the identity percentages. These identity values dropped to about 30% and 28% for *Ae*. *albopictus* and *Ae*. *aegypti*, respectively, and to 19 to 16% in the different *Anopheles* species.

Apart from the different *Culex* species, the percentage of the orthologous sequence matching the *Cx*. *quinquefasciatus* sequence is below 30%. No more than 4 to 5 consecutive identical residues are observed between the *Cx*. *quinquefasciatus* sequence and its orthologs in *Aedes* species, or *Anopheles* species, respectively. Altogether, this suggests that the 30 kDa protein presents a high specificity to the *Culex* species for which genomic data are available.

### Production of the 30 kDa recombinant protein

Fractions containing purified 30 kDa protein were separated and analyzed by SDS-PAGE. A representative purified fraction is presented in S1 Fig. The recombinant 30 kDa protein has a predicted molecular mass of 27.71 kDa (249 amino acids), and in-gel SDS-PAGE a protein band at approximately 30 kDa was detected. This protein band of interest was excised from the gel and submitted to MS analysis, confirming that the detected band corresponded to the expected 30 kDa salivary recombinant protein (S1 Table). The relative abundance of the 30-kDa protein was analyzed using ImageQuant TL software and results indicated that the purity of the protein was greater than 80%, which was considered sufficiently pure for ELISA experiments (S1 Table). Immunoblots showed the 30kDa recombinant protein as the main immunogenic component within the recombinant solution (S2 Fig).

### Study population characteristics and entomological data according to the study sites

Population demographic data (gender ratio and age structure) and entomological data are presented for each study site in Table 1. The study population comprised 223 children aged from 1 to 14 years (mean age = 7.23; 95% confidence interval [CI] [6.77–7.70]). A statistically significant difference in the mean age of the children was observed between study sites (*p* = 0.022, Kruskal–Wallis test), and two-by-two comparison using Dunn’s test indicated that the mean age was only significantly different between children from Dar-es-Salam and N’Gattakro (adjusted *p* = 0.018, Bonferroni method). The population age structure was significantly different between the four sites (*p* = 0.021). Overall, most children were in the 5 to 9 year age group, except in N’Gattakro where a higher number of children were between 10 and 14 years old. Very few children aged 10–14 years were recruited in Dar-es-Salam (*n* = 4), representing only 8% of the total number of children. The gender ratio was not significantly different between the study sites (*p* = 0.640, Pearson’s chi-squared test).

**Table 1:** Study population characteristics and entomological data according to the study sites.

	Dar-es-Salam	Kennedy	N’Gattakro	Petessou	Total	*p*
**Demographic data**						
*n* (%)	47 (21.07)	57 (25.56)	59 (26.46)	60 (26.91)	223	
Age, mean ^a^ (95% CI)	6.23 (5.44–7.02)	7.38 (6.4–8.35)	8.26 (7.34–9.18)	6.88 (5.94–7.84)	7.23 (6.77–7.70*)*	0.022
Age group ^a^ (%)						
[1–4]	14 (29.79)	14 (24.56)	11 (18.60)	20 (33.30)	-	
[5–9]	29 (61.70)	26 (41.61)	20 (33.90)	23 (38.30)	-	0.021
[10–14]	04 (08.51)	17 (29.82)	28 (47.50)	17 (28.30)	-	
Gender ratio (male/female)	0.95 (23/24)	0.9 (27/30)	1.27(33/26)	1.14(32 /28)	1.06 (115/108)	0.640
ITNs usage rate (%) ^b^	55.3	49.1	64.4	63.3	58.3	0.787
**Human biting rate (BHN)**						
*Culex quinquefasciatus*	11.7	9.8	9	0.2	**-**	<0.001
*Culex annulatus*	0.1	0.1	0	0.1	**-**	0.98
*Culex decens*	0.6	0.4	0.6	0.1	**-**	0.673
*Anopheles gambiae*	0.4	12	2.2	35.2	-	<0.001

a: years

b: insecticide-treated net

c: bites/human/night

The rate of insecticide-treated net (ITN) use was moderate, ranging from 49.1% to 64.4% in Kennedy and N’Gattakro, respectively. There were no statistically significant differences between sites (*p* = 0.787). Entomological catches showed the main anthropophagic mosquito species that were active during nighttime in each site (Table 1). *Culex quinquefasciatus* was the main mosquito species caught in N’Gattakro, and Dar-es-Salam, with a human biting rate (HBR) of 9 and 11.7 bites/human/night (BHN), respectively. Other *Culex* species were also collected but in a much lower proportion (*Cx*. *annulatus*, *Cx*. *decens*, and *Cx cinereus*). Only four *Culex* mosquitoes (two *Cx*. *quinquefasciatus*, one *Cx*. *decens*, and one *Cx*. *cinereus*) were captured in Petessou village. *Anopheles (An*.*) gambiae* was also recorded in the study sites and had the highest HBR in Petessou (35.2 BHN), while almost no *An*. *gambiae* was collected in Dar-es-Salam (HBR = 0.4 BHN). In Kennedy, *An*. *gambiae* was the main species caught (12 BHN) but *Culex quinquefacsitus* HBR was high and similar to those from N’Gattakro (9.8 BHN). According to entomological data, Petessou can thus be considered as a control village with very low exposure to *Culex*, and N’Gattakro, Kennedy, and Dar-es-Salam as the highly exposed sites.

### IgG responses to *Culex quinquefasciatus* SGE and the 30 kDa recombinant protein in children according to study sites

**Fig 3:**
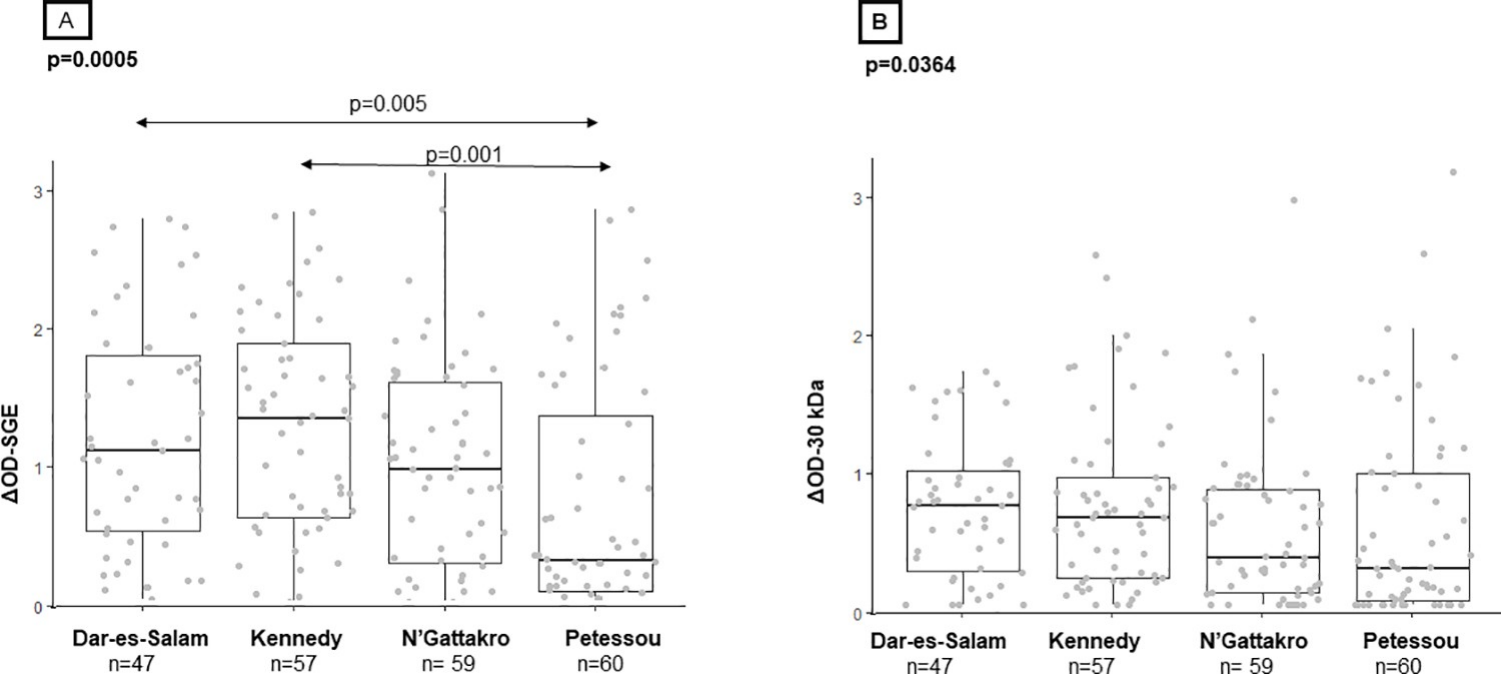
IgG responses to *Cx*. *quinquefasciatus* SGE and the 30 kDa recombinant protein in the four study sites. Each dot represents an individual and box plots indicate the median value, 25th and 75th percentile IgG response (ΔOD) against SGE (A) and the 30 kDa (B). The number of processed sera is indicated below (*n*). Significant *p-values* are indicated above the plots.

First, we tested the specific recognition of the SGE and of the recombinant protein in sera from individuals exposed to *Culex* bites. We also compared the IgG responses specific to both salivary antigens in individuals according to the site. Children from sites with high exposure to *Culex* (Dar-es-Salam, Kennedy, and N’Gattakro) and from site with low exposure (Petessou) presented a wide range of IgG responses to SGE (ΔOD-SGE; Fig 3A) and to the 30 kDa recombinant protein (ΔOD-30 kDa; Fig 3B). The median level of SGE-specific IgG was significantly different between sites (*p* = 0.0005). Running two-by-two comparisons test with Dunn’s test, the median level of SGE-specific IgG was statistically higher in Dar-es-Salam and Kennedy than in Petessou village (*p* = 0.005 and *p* = 0.001, respectively). No statistical difference was observed between N’Gattakro and Petessou (*p =* 0.290*)*. The level of IgG response to the 30 kDa recombinant protein differed significantly also between the four study sites (*p* = 0.0364), but no significant difference was noted between sites after a two-by-two comparison with Dunn’s test (adjusted p>0.05 for all comparisons). The number of participants in each village was quite small, so we estimated the precision of the two-by-two comparison tests with post hoc power analysis. The statistical power of the analysis on the IgG response to the SGE ranged from 38,9% (Petessou- Ngattakro) to 93,4% (Petessou-Kennedy) while it ranged from 5,4% (Petessou-Ngattakro) to 25,4% (Petessou-Kennedy) for the recombinant protein.

No significant differences in specific IgG responses were noted between children from the three sites with a high exposure to *Culex* (Dar-es-Salam, Kennedy, and N’Gattakro) using SGE (*p* = 0.134) or the 30 kDa recombinant protein (*p* = 0.098) as antigen source.

### IgG responses to *Culex quinquefasciatus* SGE and the 30 kDa recombinant protein in children according to the level of exposure to study sites

**Fig 4:**
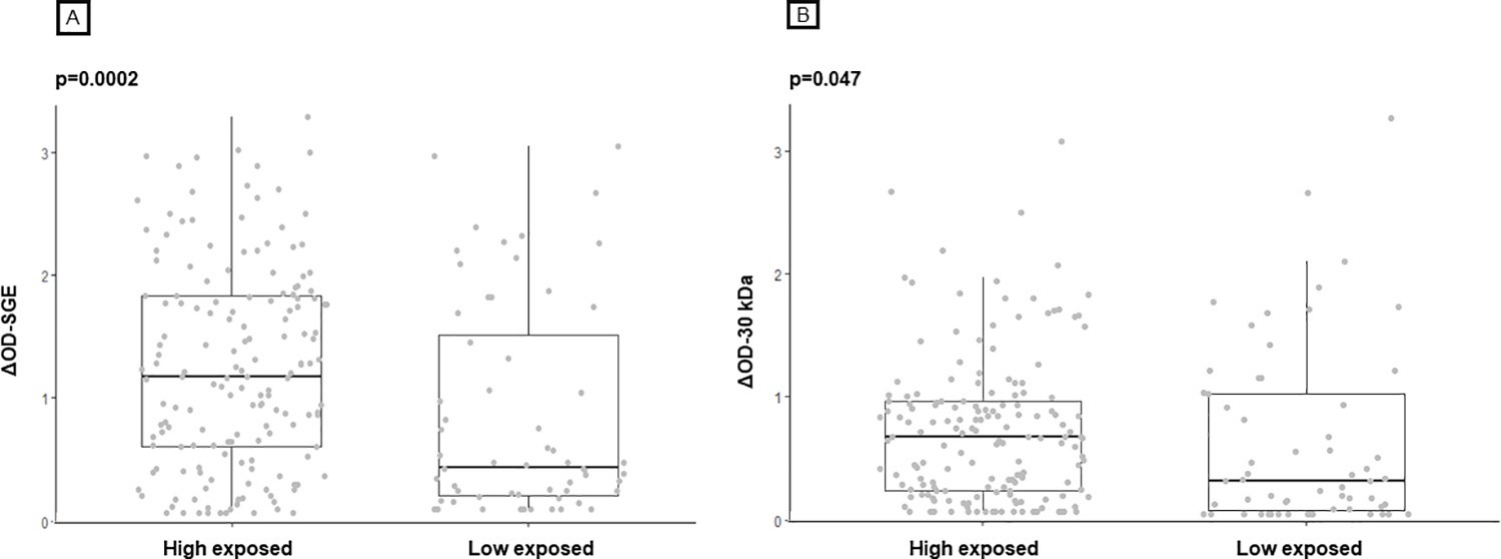
IgG responses to *Cx*. *quinquefasciatus* SGE and the 30 kDa recombinant protein according to level of exposure of sites. Each dot represents the an individual and the box plots indicate the median value, 25th and 75th percentile IgG response (ΔOD) against SGE (A) and the 30 kDa (B). The number of processed sera is indicated below (*n*). Significant *p-values* are indicated above the plots.

To assess the capacity of the IgG responses to discriminate high exposure and low exposure to *Culex* bites, we compared the level of specific IgG responses between individuals living in sites with high exposure to *Culex* (Dar-es-Salam, Kennedy, and N’Gattakro) and from the control village (Petessou with low exposure to *Culex*). The levels of IgG response to SGE (Fig 4A) and the 30 kDa protein (Fig 4B) were significantly higher in children with high exposure to *Cx*. *quinquefasciatus* bites than in children from the site with low exposure (*p* = 0.0002 and *p* = 0.047, respectively).

### IgG responses to SGE and to 30 kDa recombinant protein according to socio-demographic factors

The specific IgG responses were analyzed according to major socio-demographic characteristics (age, gender, ITNs use) and sites to identify those that could be associated with the anti-saliva IgG responses. After univariate analysis, all covariates with a *p-value* lower than 0.20 were retained in the final multivariate analysis. The results of the univariate analysis showed that age (considered as continuous or categorical variable) and gender were not associated with IgG response to SGE and to the recombinant protein (Table 2). There was an association between specific IgG responses and study sites, and the anti-30 kDa IgG response was significantly negatively associated with the use of ITNs.

**Table 2:** Univariate analysis of IgG Ab level (median) against SGE and the 30 kDa protein.

	*n*	Median of IgG to SGE	*p*	Median of IgG to 30 kDa	*p*
**Age group** (years)			0.76		0.21
[1–4]	59	0.936	Reference	0.417	Reference
[5–9]	98	0.841	1	0.445	1
[10–14]	66	1.026	1	0.645	0.27
**Gender**			0.756		0.315
Male	115	0.989		0.594	
Female	108	0.854		0.411	
**ITNs use**			0.06		**0.047**
No	93	1.063		0.581	
Yes	130	0.830		0.430	
**Sites**			**0.0005**		**0.036**
Petessou	60	0.332	Reference	0.268	Reference
Dar-es-Salam	47	1.120	**0.002**	0.736	0.079
Kennedy	57	1.348	**0.0005**	0.649	0.062
N’Gattakro	59	0.989	0.1451	0.344	1

Multivariate linear regression analysis and final modeling showed that only the anti-SGE IgG response was statistically significantly associated with the study site, with higher IgG levels detected in sites with high exposure to *Culex* (Dar-es-Salam, Kennedy, and N’Gattakro) (Table 3). No association was found between sites and anti-30 kDa IgG response. The use of ITNs was negatively associated with the IgG antibody levels against the two antigens but the effect was only significant for the anti-30-kDa IgG response (estimate = -0.192 and *p* = 0.022).

**Table 3:** Multivariate analysis of specific IgG levels to SGE and to the 30 kDa in children.

	SGE		30 kDa protein	
Final model	*R*^2^ = 0.06	*p* = 0.002	Adj. *R*^2^ = 0.019	*p* = 0.022
Covariates	Estimate	*p*	Estimate	*p*
**Sites**		**0.002**		
Petessou	Reference		-	-
Dar-es-Salam	0.467	**0.003**	-	-
Kennedy	0.494	**0.001**	-	-
N’Gattakro	0.244	0.1	-	-
**ITNs use**				
No	Reference		Reference	
Yes	-0.144	0.192	-0.193	**0.022**

### Correlation between IgG level against SGE and the 30 kDa protein

**Fig 5:**
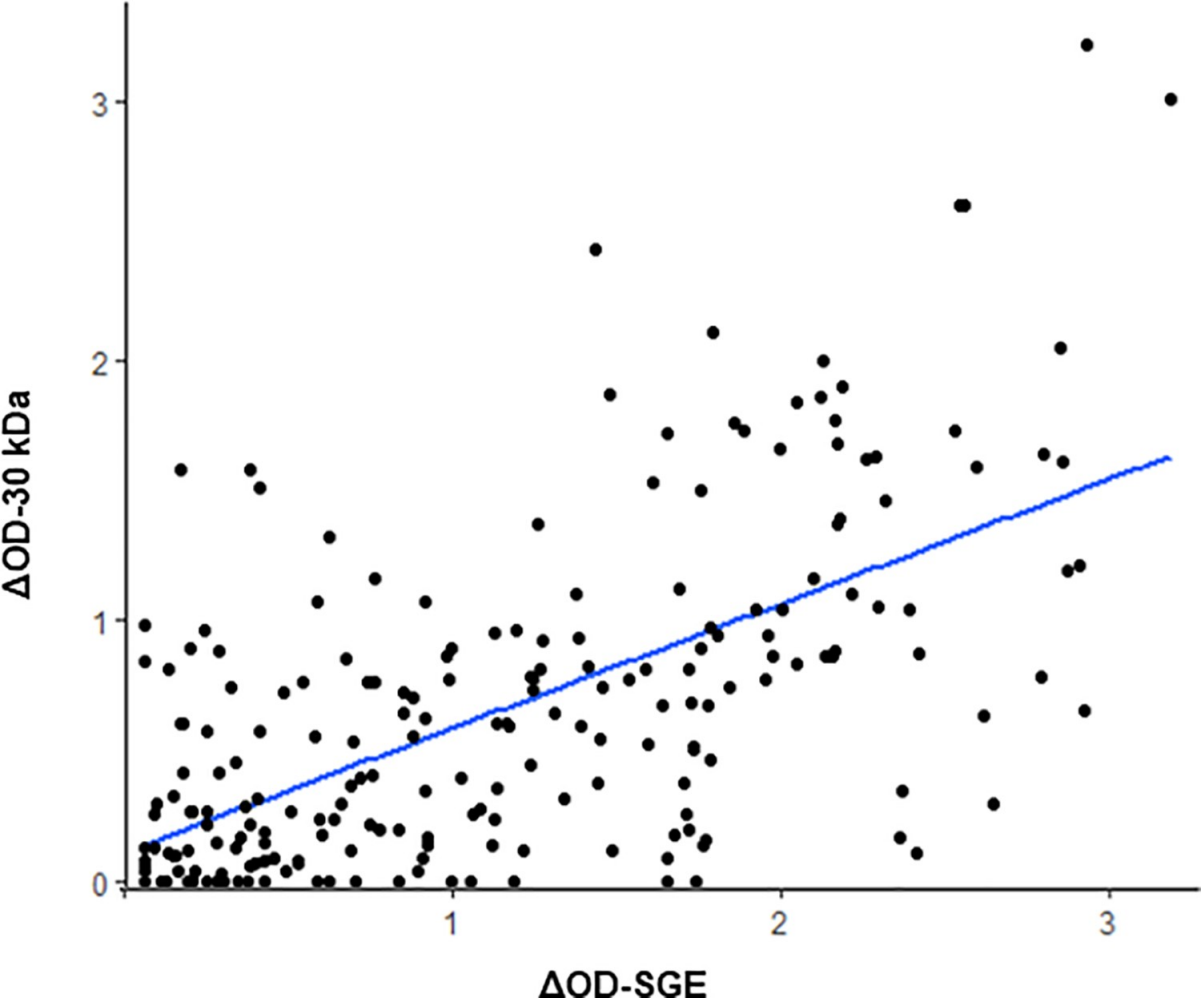
Correlation between IgG specific response to *Cx*. *quinquefasciatus* SGE and the 30 kDa recombinant protein. Scatter plot analysis of IgG responses to both salivary antigens is presented, and the ΔOD values among the 223 children are reported.

The IgG antibody levels against the two salivary antigens were compared for each child individually in the four sites using a Spearman’s rank correlation test, and the corresponding *p-values* were determined (Fig 5). A moderately significant positive correlation between the two IgG responses was found when all sites were considered together (r = +0.63, p<0.0001).

## Discussion

We investigated the Ab responses to salivary components of *Culex quinquefasciatus* in children differently exposed to *Culex* bites in order to assess whether the specific IgG responses represent a serological biomarker of human exposure to *Culex* bites. We analyzed IgG responses both to whole SGE and to the 30 kDa salivary recombinant protein. This protein belongs to the 30 kDa family and orthologs have been described in *Aedes* and *Anopheles* mosquitoes [23]. BLAST multiple alignments indicated that this protein has a low identity (<40%) and a low number of contiguous identical residues (*n*<5) with orthologs retrieved from *Aedes* or *Anopheles* mosquitoes, suggesting low cross-reactivity. The high sequence identity observed between the *Cx*. *quinquefasciatus* 30 kDa salivary protein and orthologs in two others *Culex* species suggest that the IgG response specific to the recombinant protein could be a biomarker to assess the exposure to various *Culex* species. Nevertheless, the lack of published genomic data from others *Culex* species limits the comparison.

We detected a mild-to-high reactivity to whole SGE and to the 30 kDa recombinant protein among the children, thus indicating antigenic properties in naturally exposed population to *Culex* bites. We then compared the IgG levels between children living in sites with high exposure or low exposure to *Culex* mosquitoes. Significantly higher IgG response to SGE and to the 30 kDa recombinant protein was detected in children living in sites with a high exposure to *Culex* (all “high exposed” sites taken together). The heterogeneity of SGE-specific IgG levels within the same site might reflect heterogeneous exposure to *Culex* bites. The intensity of exposure in a given population living in the same area can obviously vary between individuals. Indeed, *Culex* abundance is dependent on the presence of breeding sites and on the distance of these sites to the household. Biting intensity may be also related to human individual characteristics such as the attraction of mosquitoes, personal protection to avoid bites, and activities (indoor, outdoor).

Unexpectedly, children from Petessou, the control village where very few *Culex* were caught during entomological studies, presented significant and varying levels of IgG to SGE and to the 30 kDa protein. *Anopheles* mosquitoes were the main *Culicidae* species reported in this site, and we cannot exclude cross-reactivity with ubiquitous proteins present in the saliva of other mosquito genera, including *Anopheles*. This can be a reason for much higher antibody response to SGE than to 30 kDa protein in Kennedy, where both *Culex quinquefasciatus* and *Anopheles gambiae* were captured in high number. Entomological catches were carried out at night-time, which is not suitable for detecting the presence of *Aedes* mosquitoes that were probably also present in the sites but most active during the day-time. In addition, the HLC technique may lack sensitivity in a low exposure context that may result in an under estimation of *Culex* HBR in Petessou.

Nevertheless, children from any of the higher exposed sites to *Culex* bites had significantly higher IgG levels against SGE compared with children from Petessou (with low exposure), suggesting that an anti-SGE IgG response may help determine exposure to *Culex* bites. On the contrary, the anti-30 kDa IgG response was lower in the control village compared with any of the sites with high exposure to *Culex* but the differences were not significant. The low number of participants limited the power of statistical analysis when study sites are considered individually, particularly for the IgG response to the recombinant protein. We also showed that the IgG response to SGE or to the 30 kDa recombinant protein was similar between the three sites where entomological data reported comparable HBR for *Culex*. Global correlation analysis showed a moderate correlation between the two IgG responses.

We assessed different factors related to the IgG response with univariate and multivariate analysis. According to age and gender, no significant difference was noted between the median levels of anti-SGE and anti-30 kDa protein IgG, suggesting that neither age nor gender affect the level of specific IgG responses in the present study. This is surprising because the immune response to specific antigens is known to be acquired progressively with age. The absence of an association with age in our study suggest Ab responses to these salivary antigens may not be cumulative over time but wane rapidly. Previous studies showed the short half-life of anti-saliva Ab response in individuals naturally exposed to *Anopheles* [17] or *Aedes* [46] bites. This represents the main quality when monitoring exposure over time. In our study population, the age ranged from 1 to 14 years, limiting the comparison of immune responses in older groups.

*Culex* mosquitoes are most active during the night-time and the use of ITNs could have a major impact on human–*Culex* contact and thus on anti-saliva Ab responses. The use of bed nets was negatively associated with the IgG response to SGE and to the 30 kDa protein, with children who reported sleeping under a bed net having significantly lower IgG levels (univariate analysis). The rate of ITN use was similar between sites and thus may not explain the variation in specific IgG levels between sites.

Final models from the multivariate analysis indicated that IgG response to SGE depended on the study site, with a higher IgG response in sites with a high exposure to *Culex*. The use of ITNs was also found in the final model, but it did not seem to have a significant effect, whereas it was the only factor associated with the anti-30 kDa IgG response. Nevertheless, we did not consider the physical integrity of the bed nets, which may also alter their efficacy. Additional factors could also influence the Ab response, such as the history of exposure, co-infection, genetic background, or nutritional status [47–50]. Such data were not available in the present study and future epidemiological studies are needed to investigate additional factors (biological, environmental, or behavioral) that may influence the immune response to salivary antigens.

Altogether, these results suggest that the IgG response to SGE may represent a proxy of human exposure to *Culex* bites. A limitation to its use is related to the low specificity of these antigens with probable ubiquitous proteins shared between arthropods. The IgG response to the 30 kDa recombinant protein significantly varied between children with high exposure to *Culex* (taken together) and from the control village, but not when higher exposed sites are taken individually. However, the negative association between ITNs use and the IgG response to the 30 kDa can suggest that the 30 kDa protein might represent a potential biomarker candidate which can evaluate children’s exposure to *Culex* bites in a certain context. Additional studies should evaluate the 30 kDa candidate in a larger cohort of volunteer including older individuals (adults) and/or in other epidemiological contexts (higher *Culex* density). It would be also of interest to test IgG isotypes to assess if others antibody responses would be more relevant as biomarker of exposure.

*Culex* mosquitoes are vectors of numerous pathogens, parasites, and viruses of human and livestock. The availability of a serological tool evaluating individual exposure to *Culex* bites would be valuable for assessing the risk of exposure and transmission of related pathogens. Similar serological biomarkers have been developed for *Anopheles* and *Aedes* bites and have shown potential application for the evaluation of vector control efficacy. More studies are needed to assess the sensitivity of such serological biomarkers in order to detect small-scale spatial or temporal variation in *Culex* exposure.

## Supporting information

S1 Fig Assessment of purification recombinant form of the 30 kDa protein from *Cx*. *quinquefasciatus* expressed in *E*. *coli*. A total of 5 μg of the collected fraction was loaded per well. Band identity is listed in S1 Table. Standard molecular weights are indicated on the left side (MW: molecular weight, kDa).
(TIF)

S2 Fig Assessment of Human IgG response against the 30kD recombinant protein. **(A)** Scatter plots of Human IgG antibody responses against the purified recombinant 30kD protein from *Cx*. *quinquefasciatus*. Sera from six individuals per site (Marseille, Fos/Mer and Camargue) exposed to *Cx*. *pipiens* bites were tested by ELISA. Individuals with high and low level of IgG responses were found in each site. **(B)** Western blot of Human IgG antibody responses against the purified recombinant 30kDa protein from *Cx*. *quinquefasciatus*. A total of 23 μg of 30kDa recombinant protein was loaded onto a 10% SDS-PAGE gel (1-well of 7 cm). The immunoblots were performed by transferring the SDS-PAGE gel onto a nitrocellulose membrane. Sera from the same individuals were tested, diluted 1:100. The secondary antibody was diluted at 1:5000. Control anti-His-tag antibody was diluted 1:5000. The arrow and asterisks (*) indicate the position of the 30kD recombinant protein, detected by sera and anti-His-tag antibody, respectively. The number of each sample are indicated at the top of the WB. The individuals with high IgG responses against 30kDa in ELISA, were indicated in bold.
(TIF)

S1 Table Quantity of purified recombinant salivary protein identified by mass spectrometry. (TIF)
