# Supplementary figures and images for: First evaluation of antibody responses to Culex quinquefasciatus salivary antigens as a serological biomarker of human exposure to Culex bites: A pilot study in Côte d’Ivoire
Source: PLoS Negl Trop Dis. 2021 Dec 13;15(12):e0010004. doi: 10.1371/journal.pntd.0010004 (PMC8699949; doi:10.1371/journal.pntd.0010004)

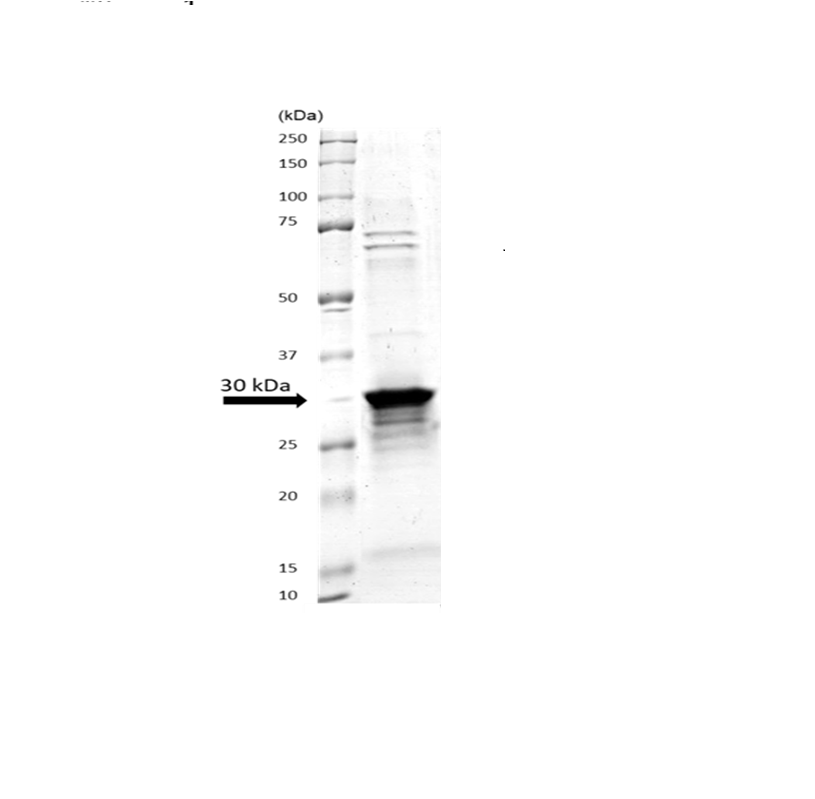

Supplement: S1 Fig — A total of 5 μg of the collected fraction was loaded per well. Band identity is listed in S1 Table. Standard molecular weights are indicated on the left side (MW: molecular weight, kDa). (TIF) [file pntd.0010004.s001.tif]

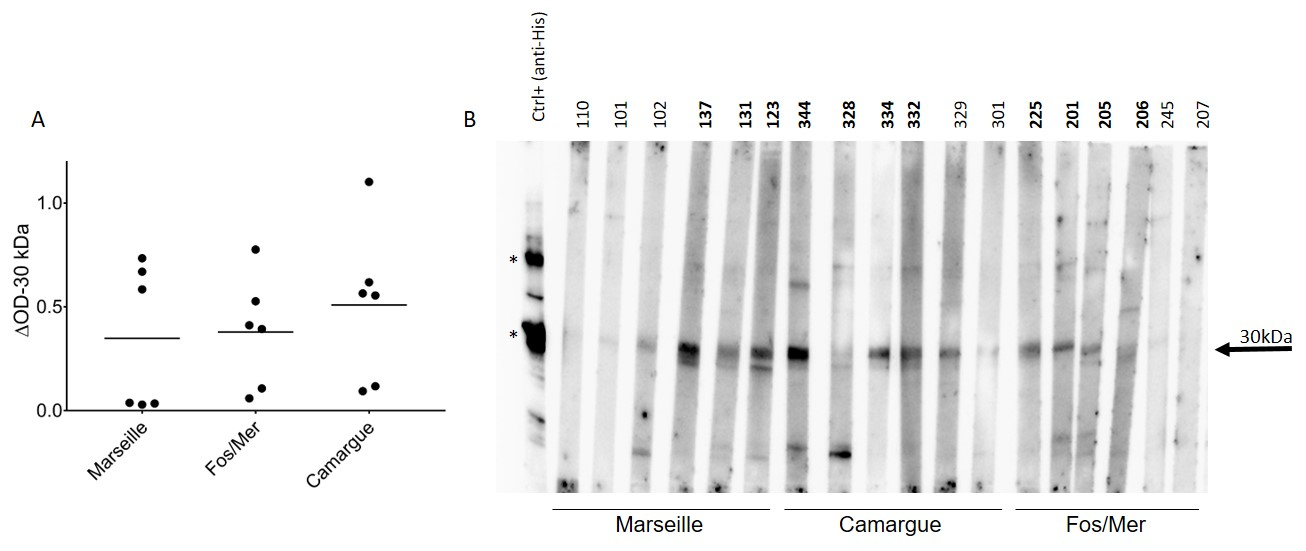

Supplement: S2 Fig — (A) Scatter plots of Human IgG antibody responses against the purified recombinant 30kD protein from Cx. quinquefasciatus. Sera from six individuals per site (Marseille, Fos/Mer and Camargue) exposed to Cx. pipiens bites were tested by ELISA. Individuals with high and low level of IgG responses were found in each site. (B) Western blot of Human IgG antibody responses against the purified recombinant 30kDa protein from Cx. quinquefasciatus. A total of 23 μg of 30kDa recombinant protein was loaded onto a 10% SDS-PAGE gel (1-well of 7 cm). The immunoblots were performed by transferring the SDS-PAGE gel onto a nitrocellulose membrane. Sera from the same individuals were tested, diluted 1:100. The secondary antibody was diluted at 1:5000. Control anti-His-tag antibody was diluted 1:5000. The arrow and asterisks (*) indicate the position of the 30kD recombinant protein, detected by sera and anti-His-tag antibody, respectively. The number of each sample are indicated at the top of the WB. The individuals with high IgG responses against 30kDa in ELISA, were indicated in bold. (TIF) [file pntd.0010004.s002.tif]

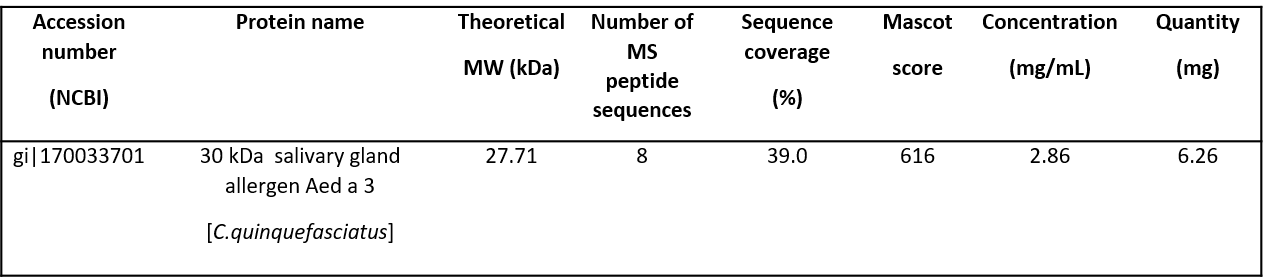

Supplement: S1 Table — (TIF) [file pntd.0010004.s003.tif]
